# Upward Bullying as Experienced by Chinese Nurse Managers: A Qualitative Study

**DOI:** 10.1155/2024/2912016

**Published:** 2024-08-28

**Authors:** Jia He, Yuhan Wang, Yangjing Wang, Xueqin Guo, Xin Li, Huan Jin, Lijuan Xiong

**Affiliations:** ^1^ Department of Otolaryngology Union Hospital Tongji Medical College Huazhong University of Science and Technology, Wuhan, Hubei, China; ^2^ Department of Urology Union Hospital Tongji Medical College Huazhong University of Science and Technology, Wuhan, Hubei, China; ^3^ Department of Nursing, Union Hospital Tongji Medical College Huazhong University of Science and Technology, Wuhan, Hubei, China; ^4^ Neurosurgery Intensive Care Unit Union Hospital Tongji Medical College Huazhong University of Science and Technology, Wuhan, Hubei, China; ^5^ Department of Anesthesiology Union Hospital Tongji Medical College Huazhong University of Science and Technology, Wuhan, Hubei, China

## Abstract

**Aim:**

To understand the current situation of upward bullying in the Chinese nursing field and explore the manifestations, reasons, and outcomes of upward bullying experienced by Chinese nurse managers.

**Background:**

Workplace bullying, a serious social problem, is characterised by recurring incidents of intimidating, aggressive, and hostile behaviour. Bullying in the nursing profession exhibits all or some of the above traits. The evidence of upward bullying by subordinate nurses against nurses in positions of authority or power is limited in China.

**Methods:**

This qualitative study was conducted with semistructured, in-depth interviews involving 12 hospital nurse managers in Wuhan, Hubei Province, between June and August 2023. The data were analysed using the Colaizzi seven-step analysis method with Nvivo 12.0 software as a support.

**Results:**

We grouped our findings into three main categories: manifestations of upward bullying; reasons for upward bullying; and outcomes of upward bullying.

**Conclusions:**

Nurse managers in China are exposed to upward bullying in many forms and for complex reasons. More emphasis needs to be given to creating a positive work environment for them to facilitate their managerial role. *Implications for Nursing Management*. This study probes the realities of upward bullying against Chinese nurse managers and highlights the need for managers to develop the skills needed to identify, manage, and prevent bullying from subordinates. By contributing to the development of interventions and strategies that address workplace bullying, this study shows promise for enhancing managerial effectiveness and improving the nursing practice environment.

## 1. Background

Workplace bullying has become a prominent global issue due to its prevalence in numerous work environments and the resulting public outcry. More than 30 years of research in various organisational settings have shown that it is one of the most complex and unpleasant occurrences in the workplace [1]. According to Giorgi et al., workplace bullying is a negative behavioural situation that individuals accept over time and find difficult to resist [2]. It is characterised by repeated intimidating, aggressive, and hostile behaviour that includes name-calling, harassment, humiliation, cold aggression, and the devaluation of job results [3]. Both harmful and antisocial, workplace bullying is a very serious problem as it can adversely affect the physical and mental health of victims, resulting in feelings of helplessness, anxiety, depression, sleep disorders, chronic fatigue, and a decline in job perception and creativity [4]. Its effects on organisations include brain drain, a tarnished image, etcetera [5].

Workplace bullying is not only found in businesses and companies but also prevalent within the nursing profession. The research found that 45% of 470 nurse educators in the United States felt they had been the target of workplace bullying in the past 6 months [6], and 53.3% of 240 nurses in China had suffered workplace bullying [7]. All of the traits of workplace bullying have been documented to various extents in the nursing literature, with nurses who experience even some of these traits exhibiting more negative coping styles, lower job satisfaction, lower staff morale, and poorer performance than their nonbullied counterparts, resulting in a potentially reduced quality of care [8–10]. Previous studies on workplace bullying in nursing have tended to focus on vertical abuse, such as toxic leadership behaviours inflicted by superior nurses on subordinates [11, 12], as well as interpersonal conflicts, known as “horizontal/lateral violence,” between nurses of the same rank [13]. However, anyone within a nursing organisation, regardless of their status, can become a victim of bullying. The victim might be a subordinate, a colleague, or a superior [14], although incidents of the latter being targets of workplace bullying are often overlooked. Managers and other high-ranking individuals are usually seen as authorities and decision-makers, and it is difficult to imagine that they can also be victims of bullying, let alone by subordinates. The modest information on bullying of nurses in managerial and executive positions, however, has suggested that subordinate nurses are the perpetrators of such behaviours [15, 16]. This type of bullying by subordinates toward their superiors is called upward bullying.

Upward bullying in the nursing workplace is defined as bullying behaviour by supervised nurses (including clinical nurses, practicing nurses, and nursing students) toward nurses in positions of authority or power (including leaders with managerial responsibilities such as nurse managers, directors of nursing, and nursing faculty) [17]. For the purpose of this study, which focused on upward bullying in the nursing clinical workplace, we considered only frontline nurse managers as the study subjects and excluded nurse educators. According to Mintzberg's theory of managerial roles [18], nurse managers are responsible for ward administration, quality improvement, service enhancement, and benefit management, and they play multiple roles such as advocate, leader, supervisor, and coordinator. However, nurse managers may struggle to perform optimally in their workplace roles due to upward bullying and find it difficult to manage within nursing organisations. Whether they choose to retreat or confront the bullying head-on [16, 17], there may be no way of preventing upward bullying from taking a toll on the body, the quality of their work, and the economy [19]. A few literature studies mentioned that factors which may contribute to the victimisation of nurse managers are being in a state of insecurity in the organisation or a shift in power dynamics within the work environment [20]. For example, a young nurse manager who has just assumed their position is more likely to be ostracised by subordinate nurses than an experienced nurse manager.

WHO has recognised bullying as a multifaceted and significant public health problem that requires close attention from families, healthcare providers, and policymakers [21]. Unfortunately, in China, there have been no reports of nurse managers' experiences of upward bullying; all the information we have is based on international literature. Since the circumstances in which nurse managers are upwardly bullied can vary due to differences in workplace culture and structure, this study uses qualitative research methods to explore the manifestations, reasons, and outcomes of upward bullying among managers within Chinese nursing organisations with the aim of finding solutions that can improve the clinical environment and enhance their managerial effectiveness.

## 2. Methods

### 2.1. Design

We adopted a descriptive, qualitative study using a semistructured interview method. Qualitative research methods, such as semistructured interviews, are well suited for data collection and analysis of real-life bullying phenomena. This work complied with Tong and colleagues' recommendations [22]. We thus adopted this method to help us identify the manifestations, causes, and outcomes of upward bullying experienced by nurse managers.

### 2.2. Sample

This study was conducted within the geographical limits of Wuhan, Hubei Province, China. Purposive sampling was used to recruit 12 nurse managers from two tertiary general hospitals and one secondary general hospital. Inclusion criteria were the following: (a) registered nurses; (b) having responsibility for staff; and (c) providing informed consent to participate in the study. Exclusion criteria included (a) nurses not on duty due to casual leave, sick leave, maternity leave, etcetera; (b) being unwilling to participate in the study; and (c) having experienced adverse psychological impact in the past 3 months, such as divorce. The previous study excluded participants with psychological distress to minimise the influence of unrelated factors [11].

### 2.3. Data Collection

A semistructured interview guide was developed, which was preliminarily drafted on the basis of a literature review and panel discussion and finalised after consultation with a nursing management expert and two pre-interviewees. The questions are presented in Table 1.

Interviews were conducted between June and August 2023, using a one-to-one semistructured in-depth interview method to collect data. Since previous research has found that telephone contact is more convenient for respondents [23] and we wanted to accommodate the busy schedules of nurse leaders, we presented the participants with the choice of face-to-face or online telephone interviews. Before the start of each interview, we explained the study's purpose, content, and methodology to the participants, assured them of the confidentiality of their information, and obtained their informed consent. We used an outline to guide the main line of inquiry, adjusting the questions flexibly according to the interviewee's responses. At all times, we avoided directional and inducing questions and encouraged the interviewees to express their true feelings about upward bullying. We stopped collecting data when information saturation was reached and no new concepts emerged [24].

### 2.4. Data Analysis

The data were analysed using Colaizzi's seven-step method [25]. All interviews lasted from 24 to 36 minutes, with an average of 30 minutes, and the audio recordings were converted to text within 24 h of the interviews to create separate documents. First, the information was read independently and repeatedly by two researchers to extract relevant and meaningful statements. Next, key information was distilled and summarised so as to identify common characteristics among the statements. We then formed themes from the data and presented the themes in relation to the phenomenon under study, while also identifying responses that indicated similar points of view. Finally, the results were returned to the interviewees to verify the authenticity of the content. In cases of disagreement during data analysis, we reviewed the notes and recordings, and if there was still disagreement, the respondent was re-interviewed until agreement was reached. To assist in analysing the data, Nvivo 12.0 software was used.

### 2.5. Ethical Considerations

The study was approved by the Ethical Committee of Tongji Medical College, Huazhong University of Science and Technology (No. S044). Our work was conducted in accordance with the Declaration of Helsinki. All the people participating in the research voluntarily agreed to be interviewed and received information about the study as well as a letter of commitment to confidentiality signed by the research team. In addition, the participants all signed an informed consent form in accordance with said participation which indicated their right to discontinue their participation in the study at any time.

## 3. Results

### 3.1. Demographic Characteristics of Participants

All 12 respondents in nursing management positions met the inclusion criteria. All participants were female, all had the title of nurse-in-charge, and all had a bachelor's degree or higher. Among the 12 participants, 11 were the head nurse of the unit and one was the deputy director of the nursing department, and they all managed an indeterminate number of staff. The age range of the participants was between 33 and 41 years with a mean age of 36.6 years.  Table 2 presents the general demographics.

### 3.2. Categories and Themes Emerging from the Data

The interview data provided rich details of how and why nurse managers were bullied by those in subordinate positions during their current period of employment. Based on their narratives, we grouped the data into three categories from which we distilled and summarised eight themes, as shown in Table 3. In the sections that follow, we expand on these categories and themes and provide direct quotations (translated from Chinese) from nurse managers to support our inferences.

#### 3.2.1. Manifestations of Upward Bullying

Manifestations of upward bullying refer to outward forms of bullying by subordinates toward nurse managers expressed in behaviour, words, etc. The main forms of bullying that emerged from this study included verbal aggression, threats, and disregard for authority.

*(1) Verbal Aggression*. Respondents indicated that they were receptive to ideas from their subordinates if the ideas were constructive and would like to work with their department members to create a better departmental atmosphere and improve the organisational climate. However, some subordinate nurses chose to express their opinions or dissatisfaction by launching verbal attacks, either in person or behind their backs. These attacks took the form of sarcasm, backbiting, and public humiliation. A head nurse said, “When I first took up my post, if there was any disagreement with the nurses, they belittled my ability to do my job and even called me “eye candy”, meaning “impressive but lacking real worth,” which I found demeaning and offensive” (N6). Another one said, “Nurses often talked privately about my lack of fairness and there was no shortage of public dislike for me” (N1).

*(2) Imposing Threats*. A few respondents claimed that some subordinate nurses would express their dissatisfaction with their job or managers by issuing threats. As a result, nurse managers were most often forced to compromise on their management style and alter their way of speaking. A head nurse who had been in management for 2 years recalled, “This really struck me. At that time, when I made new rules and corrected the young nurse's missteps, she threatened to stop my management of her by “jumping off the roof”” (N5). Another head nurse said, “Some of the nurses threatened to leapfrog me [i.e., report me] by writing confidential letters. While I know I didn't make a mistake, I still didn't want to get myself in trouble” (N10).

*(3) Diminished Authority*. Authority typically confers a certain prestige and credibility in the minds of subordinates, although not always. Respondents indicated that subordinate nurses would challenge and undermine their authority as leaders in various ways. One manager said, “She would not follow my orders or instructions and even ignored me” (N2). Another head nurse explained her situation in these words, “I was treated with indifference and [the subordinate nurse] was not supportive of my decisions; she even tarnished my image in front of other nurses and instigated opposition toward me, which made my management job more difficult” (N7).

#### 3.2.2. Reasons for Upward Bullying

Reasons for upward bullying refer to the conditions that cause or trigger bullying of nurse managers by subordinates. This study found three factors that prompted bullying: conflicts of interest, differences in position, and excessive workload.

*(1) Conflicts of Interest*. Respondents revealed that conflicts of interest, both between individuals and between individuals and the collective, was one reason for upward bullying. A new manager claimed, “The new head nurse is bound to change some of the management mode. And the nurses who have taken the “bonus” of the previous manager will have a gap in their hearts, thinking that their benefits have changed from more to less” (N3). Other respondents mentioned, “Meeting everyone's needs is difficult to balance. Nurses resent me when they notice small inequalities” (N8). One manager said, “Some people think nursing is just a job and are not willing to make compromises for the sake of collective progress, which makes my work hard” (N12).

*(2) Differences in Position*. Some respondents pointed to conflicts that arise due to differences in the scope of work between nurses and managers. The deputy director of the nursing department explained, “I am in a position where I have to consider things on a more macro level. Some nurses are not mature enough to understand my intentions, and this leads to misunderstandings” (N11). A head nurse said, “The work mentality is different. For example, there are requirements for the placement of oxygen tanks, and some nurses think it's most convenient to put them somewhere, but I will ask them to change the placement from the perspective of management, and that's when we disagree” (N4).

*(3) Excessive Workload*. One external reason for upward bullying is the heavy workload of nurses, which leads to higher physical and mental stress and emotional backlog. A head nurse sighed, “At present, there is a shortage of human resources in the department; nurses work night shifts frequently, and they are physically and mentally exhausted. As a manager, I want to schedule shifts for my staff more effectively to reduce the burden, but the workload is objective and cannot be changed” (N9).

#### 3.2.3. Outcomes of Upward Bullying

Outcomes of upward bullying refer to its effects on victims and organisations. This study grouped outcomes into two main categories: positive and negative outcomes. The following sections discuss the themes that were found within each of these categories.

*(1) Self-Reflection and Improvement (Positive Outcomes)*. Managers suggested that their experiences of upward bullying spurred them to engage in self-reflection so as to improve themselves and their practices. Specific actions included taking the initiative to improve their management skills, reinventing themselves, identifying and solving problems at work, and improving their mindset. The deputy director of nursing reflected, “Management work is not infallible, all things have room for improvement. Why people are sometimes in a bad mood is something I need to think about, and I will take the time to work on myself” (N11). One manager said, “I have not only asked senior nurse managers for advice on how to manage and communicate with my subordinates, but I have also taken relevant courses and referred to relevant books” (N2).

*(2) Sadness, Anger, Loss of Trust, Etcetera (Negative Outcomes)*. The negative effects of bullying behavior are unavoidable. On the individual side, the above behaviors can bring anger, sadness, and other emotions to nurse managers, depleting emotional resources; increase the pressure of managing and coordinating work; and also affect the establishment of managers' personal authority, damaging leadership charisma and trust between supervisors and subordinates. On the organisational side, bullying behavior can affect the atmosphere of the department and undermine team cohesion. As two managers noted wearily, “It does come with a sense of loss and chills” (N12, N7). Another manager stated quite plainly, “If the abuser is someone who is a strong communicator and is used to complaining to others, it can disrupt the team atmosphere and reduce my managerial effectiveness” (N8).

## 4. Discussion

In this study, we used qualitative research techniques to explore nurse managers' experiences with upward bullying in Chinese nursing organisations. The responses of 12 managers allowed us to determine that upward bullying does indeed exist in these organisations, and that there are both similarities and contrasts with findings from other regions. As WHO argues, bullying is a multifaceted and significant public health problem, which is detrimental to creating a healthy, person-centred work environment in healthcare organisations.

When asked if they were aware of upward bullying, 75% of the respondents paused to reflect on the question. They indicated that they were more familiar with “horizontal violence” as well as “downward bullying” and that they were unaccustomed to viewing themselves from the perspective of a victim with respect to workplace bullying. After being informed of the definition of upward bullying, managers acknowledged that they did struggle with interpersonal conflicts with their subordinates, which caused some distress in their work environment and posed an obstacle to work advancement. In China, society is characterised by differentiated positions, and managers can usually achieve goals and solve problems quickly by relying on the authority invested in them [26]. However, this study shows that the ability of nurse managers to cultivate the charisma needed of a manager and to develop rapport and trust with subordinate nurses is significantly impeded by various forms of upward bullying, including verbal abuse, threats, fabrication of ratings, and deliberate negative feedback [15].

In line with the results of this study, the reported causes of upward bullying in the literature include an inflexible and heavy workload, differences in education and mindset, as well as jealousy and other emotions [17, 27]. It is worth noting that one of the themes emerging from the data in this study was “conflicts of interest,” which were manifested mainly in the differences in benefits that individuals receive and in the interests between individuals and organisations. There is a consensus among scholars that bullying is easier to comprehend when the organisational context in which it occurs is taken into account [28]. Since collectivism is one of the key social values of Chinese culture, individuals place high priority on relations with others and with the collective [29]. Chinese organisations guide and run teams with the goal of integrating individuals into the collective, and many individuals will voluntarily sacrifice their individual interests for the sake of the collective if harmony cannot otherwise be achieved [30]. With respect to organisational advancement, nursing managers want to promote their teams and units, which invariably mean adding to the burden placed on staff nurses. In response, staff nurses will sometimes accuse the nurse manager of implementing measures that violate their interests and engage in a number of bullying tactics to vent their frustration.

In addition, our findings are consistent with other studies showing that bullying has a variety of detrimental repercussions on nurse managers, including dwindling emotional resources, decreased productivity, poor physical health, and even jeopardised patient safety [15, 31]. When managers are the target of subordinates' bullying, they themselves can be in a state of information disconnect, unable to effectively coach and manage their team. Struggling to take control of the whole team, they can become reactive, with effects that include organisational mismanagement and higher propensity to quit [15, 17].

Interestingly, the Chinese respondents in this study retained a positive attitude despite the upward bullying. They continued to pursue additional education, behave positively, and serve the public in an effort to strengthen their personal charisma as the “head” of the unit. Research indicates that the types, causes, and manifestations of bullying in the nursing field have undergone staged changes over the last 4 decades, with nursing management having had a role to play in addressing the problem of bullying [32]. Nurses who take on management roles may be able to build up personal and organisational resources by taking positive action, as did respondents N11 and N2. Taking a proactive stance allows them to gain experience in managing upward bullying issues that are closely related to their own, as there is limited information on upward bullying in the Chinese nursing field. This article can help nurse managers to understand the ways in which upward bullying behaviour manifests itself in the profession and, most importantly, prompt managers to develop the necessary skills to identify, manage, and prevent bullying from subordinates in order to maintain their managerial effectiveness.

While antibullying laws and zero-tolerance campaigns have been tried in some countries to enable nurse managers to respond more adequately to the devastation caused by bullying, they fail to address the most fundamental problems [33]. According to Parchment and Andrews [15], nurse managers with authentic leadership styles report less bullying. Therefore, managerial training programs oriented to leadership development and improving interpersonal skills may be one way to strengthen the leadership skills of nurses and reduce bullying. However, addressing upward bullying in nursing is not solely the responsibility of nurse managers. Supervised senior nurses have a wealth of workplace experience and interpersonal communication skills that allow them to bridge the communication gap with managers and serve as a benchmark for younger nurses in China. Therefore, senior nurses also need to demonstrate leadership in their professional roles and show respect for their supervisors, colleagues, and the profession [32]. By role-modelling appropriate behaviour, they can help to reduce the incidence of upward bullying.

## 5. Limitations

The participants in this study were all from hospitals in Wuhan, Hubei Province, and further research should be conducted to expand the representativeness of the sample. Moreover, the study participants were all female, and whether gender is a factor in upward bullying remains to be explored. Finally, qualitative research methods cannot determine the true frequency of upward bullying in the Chinese nursing field.

## 6. Conclusion

In China, upward bullying is a reality in the field of nursing, with nurse managers encountering various degrees and types of bullying just as they do in other countries. In addition to objective changes in workload and psychological displeasure, other factors also trigger this type of bullying behaviour. Some Chinese nurse managers are strong-willed and responsible. They are able to turn a negative situation into an opportunity for growth. Creating a positive work environment for them that facilitates management, however, is still something to be emphasised.

## 7. Implications for Future Nursing Management Research

Limited evidence exists of upward bullying in the field of nursing, especially in the Chinese context. This study can contribute to an understanding of why such bullying occurs and what its outcomes entail. Given that bullying behaviour is linked to turnover, we have concerns that the continuation of upward bullying will have a similar impact on the retention and turnover rates of nurse managers in China, which would pose a threat to organisational cohesion and management. We suggest, therefore, that the relationship between turnover rates and exposure to upward bullying among nurse managers in China be further investigated.

It is hoped that the findings of this study will enrich future research in the fields of nursing management and workplace health in China and inform large-scale quantitative studies that investigate the issue of upward bullying. And we anticipate that this study will contribute to the development of interventions and strategies to manage and address the upward bullying of nurse managers and that these actions taken will improve management effectiveness and the nursing practice environment. In addition, the conclusions of this study prompt us to rethink the importance of professional literacy training in nursing education.

## Figures and Tables

**Table 1:** Interview questions.

(i) Please briefly introduce yourself (age, marital status, length of independent nursing practice, and nursing title)
(ii) Have you heard of the term “upward bullying”? What do you understand by “upward bullying”?
(iii) Have you ever been the victim of upward bullying or witnessed it among others since becoming a nurse manager?
(iv) If you have been exposed to upward bullying, what did you specifically witness or experience?
(v) If you have been the victim of upward bullying, why do you think it occurred?
(vi) How has upward bullying in the workplace affected you? How do you feel about it?
(vii) What measures are in hand to help reduce the incidence of upward bullying in your workplace?

**Table 2:** Demographics of participants (*N* = 12).

Participant no.	Female (F)/male (M)	Age (years)	Education	Professional title	Position	Level of hospital
1	F	39	Bachelor's degree	Nurse-in-charge	Head nurse	Tertiary
2	F	34	Master's degree	Nurse-in-charge	Head nurse	Tertiary
3	F	33	Master's degree	Nurse-in-charge	Head nurse	Tertiary
4	F	38	Master's degree	Nurse-in-charge	Head nurse	Tertiary
5	F	37	Bachelor's degree	Nurse-in-charge	Head nurse	Tertiary
6	F	33	Bachelor's degree	Nurse-in-charge	Head nurse	Secondary
7	F	41	Bachelor's degree	Nurse-in-charge	Head nurse	Tertiary
8	F	37	Master's degree	Nurse-in-charge	Head nurse	Secondary
9	F	40	Master's degree	Nurse-in-charge	Head nurse	Secondary
10	F	38	Bachelor's degree	Nurse-in-charge	Head nurse	Tertiary
11	F	33	Bachelor's degree	Nurse-in-charge	Deputy director of the nursing department	Secondary
12	F	36	Master's degree	Nurse-in-charge	Head nurse	Tertiary

**Table 3:** Summary of categories and themes.

Categories	Themes
Manifestations of upward bullying	Verbal aggression
	Imposing threats
	Diminished authority

Reasons for upward bullying	Conflicts of interest
	Differences in position
	Excessive workload

Outcomes of upward bullying	Self-reflection and improvement
(Positive and negative)	Sadness, anger, loss of trust, etcetera

## Data Availability

The data that support the findings of this study are available from the corresponding author upon reasonable request.
